# Public Health in the United States.—III

**Published:** 1906-01-27

**Authors:** 


### PUBLIC HEALTH IN THE UNITED STATES.—III.

(*Concluded from page 278.*)

### SEWAGE DISPOSAL.

CLOSELY related to the subject of water supply is that of sewerage and sewage disposal, since the sewage consists of the water of the public water supply, with the addition of such household filth and manufacturing wastes as may be added to it by the population. Both are sanitary necessities of great importance, but the public water supply is usually introduced long before serious thought is given to the question of sewerage. It is for this reason that the percentage of the population living in sewered towns is very much less than that of the towns furnished with public water supplies. The percentage of people living in sewered towns in the United States in 1896 was 28·7, while the percentage of the total population living in towns having public water supplies was 41·6. The number of cities and towns which had introduced systems of sewerage in 1896 was 822, or less than one-fourth of the total number of cities and towns furnished with public water supplies.

The methods of disposal are various, and are necessarily dependent upon the conditions and circumstances which prevail in each case. In the case of cities situated on the sea coast, or in large bays, harbours, or tidal estuaries, the sewage is usually discharged into sea water without treatment. This is the method pursued at Portland, Salem, Lynn, Gloucester Boston, Providence, New York, Baltimore, and San Francisco The greater portion of the sewage of Boston is retained, and only allowed to discharge on each outgoing tide, while that of the remainder of the city, and of 14 neighbouring cities and towns, is discharged into deeper water continuously. Most large cities situated upon the great lakes and rivers discharge their sewage directly into those bodies of water, as at Philadelphia, Cincinnati, St. Louis, Albany, Minneapolis, St. Paul

Washington, Buffalo, etc. In some instances, these bodies of polluted water are the sources of water supply of cities situated at lower points on the same stream. It was this condition which caused serious epidemics of typhoid fever at Chicago, Philadelphia, etc., in some of which radical changes in the methods and sources of supplying water have been introduced as a consequence.

Disposal upon land has been conducted with entirely satisfactory results in several cities and towns, but the number of such places is not large. Chemical precipitation is also conducted in a few places, the most notable instance being the City of Worcester, having a population of about 100,000.

### School Hygiene.

Laws have been enacted in several States, placing the sanitary supervision of school-houses under the control of definite authorities empowered to act, and to make or to cause to be made, such changes as are necessary to place these buildings in a proper sanitary condition. In some States this authority has been granted to Boards of Health, in others to the school authorities, and in others to special State or district police officials. Physical training has in recent years been introduced in the schools of all large cities, and the tendency is to place this important branch in the hands of educated instructors.

The plan of making regular medical inspections of the pupils attending the public schools, so far as the United States is concerned, was introduced by a communication from the Board of Health of Boston to the Mayor and City Council in 1892. In November 1894 a corps of 50 physicians was appointed for as many districts in the city. These inspectors were instructed "to visit the schools daily in the morning, and to examine all pupils who complain or appear to the teachers to be ill." In the examination of throats wooden tongue depressors are used, to be destroyed after each single examination. These inspectors are also authorised to visit the homes of the children thus found to be ill, and to see that proper precautions are taken at home for preventing the spread of disease. During the 14 months ending December 31st, 1895, 16,790 pupils were examined, 10,337 of whom were found to be ill, and of these 77 had diphtheria, 28 scarlet fever, 116 measles, 28 chicken-pox, 69 pediculosis, 47 scabies, 47 mumps, 33 whooping-cough, and 8 congenital syphilis. The remainder were suffering from a variety of other diseases, and many of them were found to be too ill to remain in school during the day.

### Municipal Hygiene.

The necessity of public sanitation, judiciously administered by some well-organised municipal authority, increases in proportion to the increase in density of the population. Hence every city and nearly every large town of 10,000 inhabitants or more, in the United States, now has its board of health or health department, organised for the purpose of providing for the protection of the public health of the citizens living within the limits of such municipality. For the smaller towns, villages, and rural districts which comprise at present the greater part of the population of the United States, the laws are much more variable. The boards of health or health commissioners of cities are usually appointed by the city government, while those of the towns are more commonly elected by the people, in the same manner with other important local officers.

The principal duties of municipal boards of health are the following:—The management and control of infectious diseases, including notification, isolation, disinfection, vaccination, and the supervision of infectious disease hospitals; the inspection and abatement of local nuisances; the sanitary inspection of the food supply, and specially that of milk, provisions, and animals used for food; street cleaning; the collection and disposal of ashes, garbage, and refuse; tenement house inspection; medical inspection of schools; supervision of foundlings, infant asylums, and lying-in hospitals; inspection of plumbing; inspection of bakeries; inspection of barber-shops; registration of vital statistics, and supervision of burials; care of public bathing establishments; regulation of offensive trades; regulation of stables; supervision of the municipal water-supply, and the system of sewerage and sewage disposal.

### Industrial Hygiene.

A very large proportion of the population of the United States is engaged in useful occupations, the total number of such employed persons amounting to many millions. Some or these occupations or industries, and specially those which are conducted indoors, have a decided effect on the health of those who are employed. The special industries in which operatives are subjected to harmful influences are mining, cotton and woollen manufacture, paper-making (specially in the department of rag sorting and cutting), stone-cutting, grinding and polishing of steel implements, match-making, wool and hair sorting and hide-cleaning, the manufacture of poisonous colours and dyes, pottery, and all manufactures into which lead enters as a component part (painting, plumbing, type-setting, glazing, &c.). To these may be added certain industries and occupations in which the employed are specially liable to serious accidents involving the loss of life, limb, or sight. But little organised work has been accomplished thus far in America in this important direction, other than that which has been provided for by the enactment of certain laws requiring factory inspection by special officials appointed for the purpose. The chief danger in these occupations is that which results from the inhalation of dust, either of an irritating character, as in the case of file-cutting and needle-grinding, or of a specific poisonous nature, such as is illustrated by the dust of infected foreign hides and hair, and that of the floors of workshops infected with the expectoration of tuberculous workmen. Legislation is very much needed providing for a careful investigation of the conditions to which operatives are subjected in harmful employments, the causes of the evils, and the best means of applying the proper remedy.

### Rural Hygiene.

In the United States the population occupying the rural districts is still largely in the majority and probably amounts to nearly 50 millions. There are few occupations in which hygiene is more neglected than that of the farmer. One defect in the hygiene of the rural population of the United States is that of badly selected or badly cooked food. Notwithstanding the great abundance and variety of food, both animal and vegetable, which is produced on the well-tilled farms of New England and on the western prairies and fertile fields of the south, it is undoubtedly true that the food of the farmer is less varied and less wholesome than that of the urban population. In the more densely populated States it often happens that the better and the more nutritious products of the farm (eggs and the products of the dairy) are sent to the markets of the neighbouring cities, while the farmer's family is fed on a limited and less nutritious diet. The chief defects of the diet of the rural population may be stated as follows:—Too exclusive use of fried food; salt meat to the exclusion of fresh meat; exclusive use of fine wheat flour in the place of the coarser and more wholesome sorts of meal and flour which were largely in use half a century since. Pork in some form is used as food by a very large part of the population of temperate climates, and when it is the product of healthy animals it is a nutritious and wholesome food for all who are accustomed to a life of toil, and for those who have naturally vigorous constitutions. Swine, however, are subject to many diseases, some of which, specially those of a parasitic nature, are communicable to man. It has been observed that those animals which are fed on the offal and garbage of cities are infected in a much greater degree than those which are fed on wholesome food. Tape-worm commonly has its origin in eating the flesh of swine which has been insufficiently cooked. The Government provides an ample force of inspectors at the principal pork-packing places for the purpose of securing protection to the consumers of this pork in other countries to which it is exported. But the agriculturist himself, who produces the pork upon his farm, has no protection for his family who may be consumers of the same, except that which common sense at once suggests—the thorough cooking of the meat.

### Medical Education.

A comparison of the condition of the medical education to-day in the United States with that which prevailed in the middle of the century shows a very great advance. There is a tendency to require that chemistry, botany, comparative anatomy, and other allied branches should be considered as preliminary work, and that the medical student should receive instruction in them at some college or scientific school previous to beginning his course of medical study. In the department of instruction in preventive medicine or hygiene there is an opportunity for improvement. There is a rapidly increasing demand for men to fill the places of public-health officials, chemists, and bacteriologists for this special line of work. The importance of the position of medical men in this connection is recognised by the fact that no sanitary board or organisation in any State or large city is ever established without including one or more physicians in its membership, and in very many instances the entire board consists of medical men.

The actual number of registered physicians in the United States shows an average for the whole of about 1 to 647 inhabitants, but the proportion in the different States varies greatly.

